# Status report on the quantum chemical cluster approach for modeling enzyme reactions

**DOI:** 10.1038/s42004-022-00642-2

**Published:** 2022-03-08

**Authors:** Fahmi Himo, Sam P. de Visser

**Affiliations:** 1 Department of Organic Chemistry, Arrhenius Laboratory, Stockholm University, SE-10691 Stockholm, Sweden; 2 Manchester Institute of Biotechnology and Department of Chemical Engineering and Analytical Science, The University of Manchester, 131 Princess Street, Manchester, M1 7DN UK

**Keywords:** Biocatalysis, Method development, Computational chemistry, Enzyme mechanisms, Quantum chemistry

## Abstract

The cluster approach is a very valuable technique for elucidating reaction mechanisms of enzymes. Here, the authors discuss the current status of this methodology, highlighting its strengths and weaknesses, and argue that it should be the method of choice for investigating enzymatic reaction mechanisms.

## Preface

The cluster approach is a very valuable technique for elucidating the reaction mechanisms of enzymes. Here, the authors discuss the current status of this methodology, highlighting its strengths and weaknesses, and argue that it should be the method of choice for investigating enzymatic reaction mechanisms.

Enzymes are Nature’s sophisticated catalytic machines with functions related to biosynthesis and biodegradation of natural products. Their size and complexity are daunting for computational chemists. Nevertheless, over the years computational chemists have developed an array of approaches that are able to model various aspects of enzymes’ functions and properties, at different length and time scales. These methods are so powerful these days that the computations can be used as a predictive technique that can guide experimental work.

## The cluster approach

The quantum chemical cluster approach is a technique devised to investigate reaction mechanisms and spectroscopic properties of enzyme active sites^1–3^. It relies on the use of relatively accurate quantum chemical methods that can predict bond strengths, physicochemical properties, and spectroscopic features of the active site of the enzyme. Most common approaches use density functional theory (DFT) methods and share many common features with how homogeneous catalysis and biomimetic models are calculated.

**Fig. 1:**
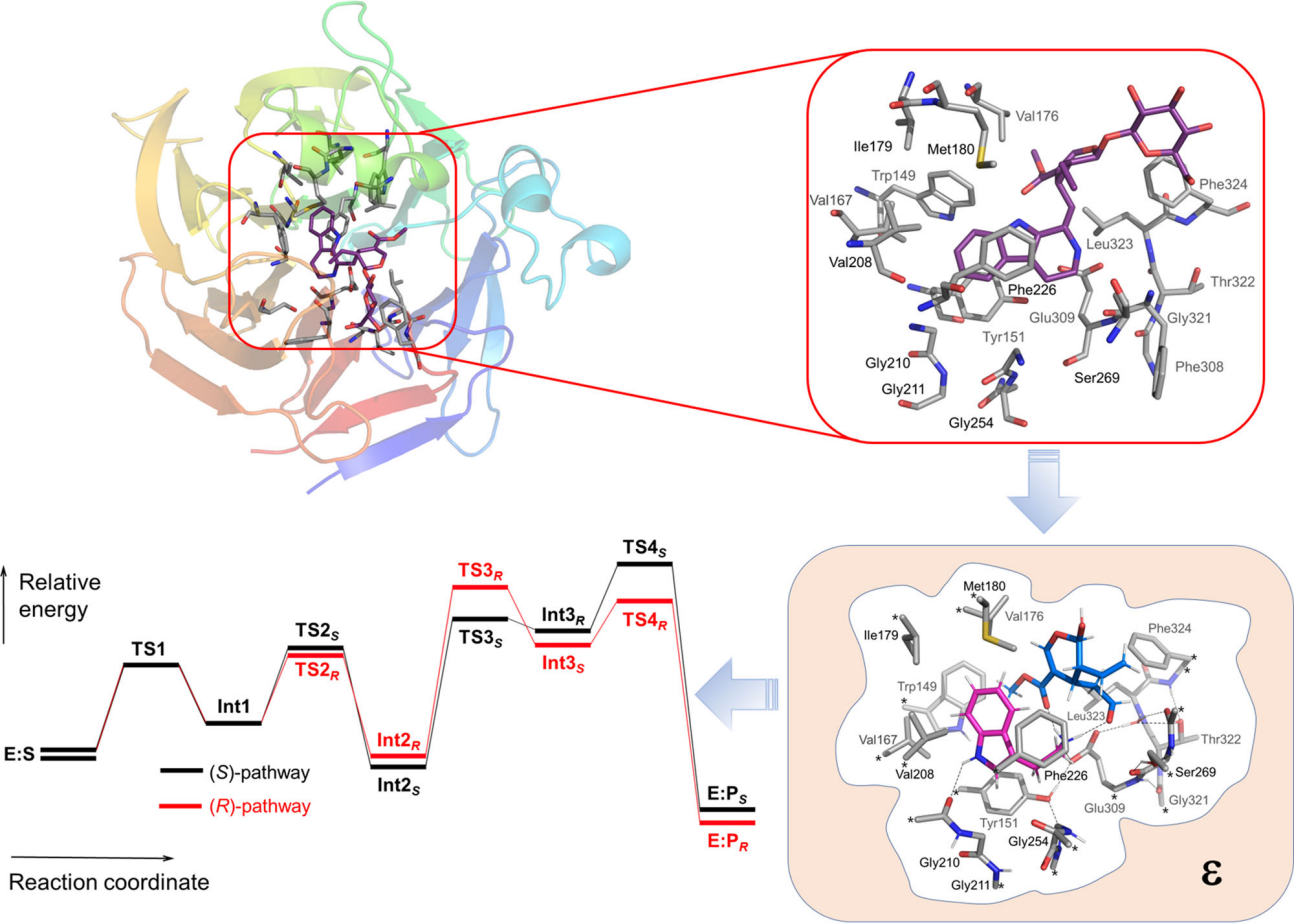
Schematic drawing of the steps involved in the cluster approach. A model of the active site is designed on the basis of a crystal structure. A number of positions at the edge of the model are kept fixed during the geometry optimizations, and the surrounding is modeled as a continuum solvent. Intermediates and transitions states are located and their energies are used to judge reaction mechanisms and elucidate the origins of selectivity.

In brief, a limited model of the active site is carved out of the actual structure and an energy profile for a possible reaction mechanism is calculated, including all intermediates (local minima) and transition states leading to products and by-products through bifurcation pathways (Fig. 1). The feasibility of a certain mechanism can thereby be judged. Furthermore, the bifurcation pathways and transition states give structural and electronic explanations of what drives the selectivity of the reaction and how it could be manipulated. The rest of the enzyme is typically modeled assuming a homogeneous polarizable surrounding, and to keep the active site architecture reasonably intact, a number of centers at the periphery of the model are usually kept fixed to their crystallographic positions.

## Size of active-site models

**Fig. 2:**
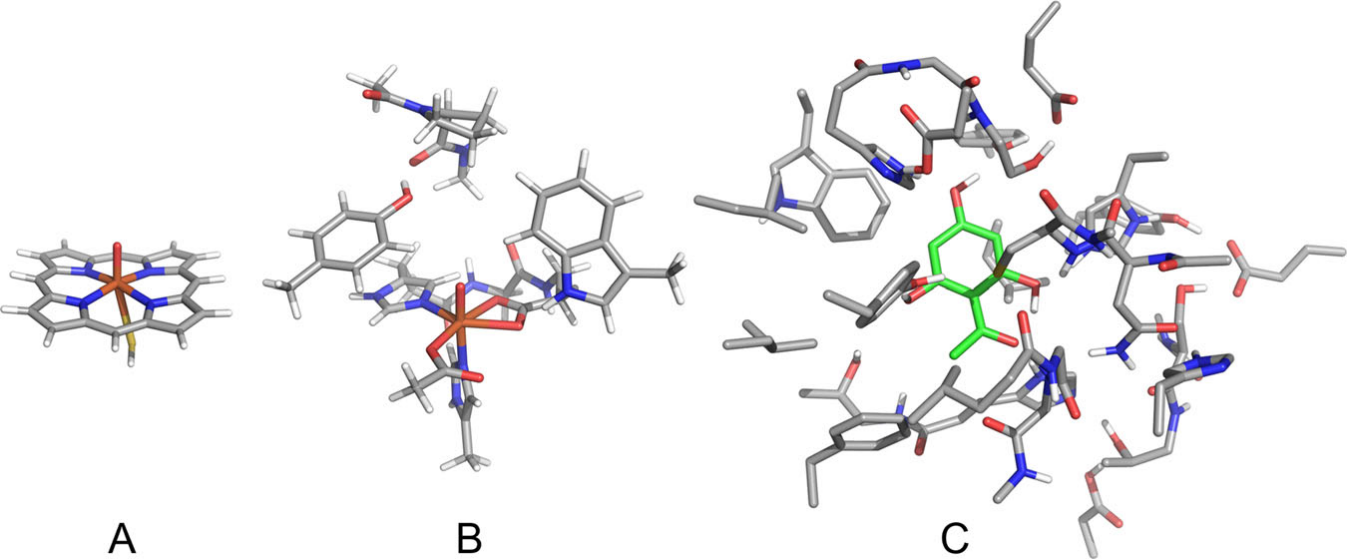
Examples of active site model sizes at different times. **A** 40 atoms: Cytochrome P450 in 2000^7^. **B** 116 atoms: Prolyl-4-hydroxylase in 2010^8^. **C** 413 atoms: Acyltransferase in 2020^9^.

Some 20 years ago, active site models were typically 30–50 atoms in size and did usually not contain many atoms beyond the first-coordination sphere of the metal center^4–6^. With the improvement of computer power, it soon became possible to treat larger models that include some part of the second-coordination sphere, which determines the substrate and cofactor positioning. These models were initially 100–150 atoms in size, while today’s cluster models typically have >300 atoms (Fig. 2)^7–9^. The larger models make of course a more accurate representation of the real system and typically include all first- and second-coordination sphere effect perturbations, including those of charged groups and key hydrogen bonding and π-stacking interactions^10^.

## Some applications

The cluster approach has been instrumental in elucidating the catalytic cycles of a large number of enzymes and has been particularly helpful in gaining an understanding of the short-lived intermediates during the reaction^1–3^. As an early example of this, we can mention the work on cytochrome P450 enzymes, where experimental studies had proposed a second-oxidant active in the catalytic cycle, namely Compound 0 or the iron(III)-hydroperoxo species, rather than Compound I or the iron(IV)-oxo heme cation radical species^11^. However, computational modeling established that Compound 0 cannot react with olefins or aromatic rings at room temperature and that Compound I was the sole oxidant in the P450s^12^.

Over the years, the cluster approach has been used to resolve experimental controversies and inconsistencies and has also guided further experimental studies. It has solved some of the most outstanding and complex problems in enzymology, such as, for example, the mechanism of dioxygen synthesis by Photosystem II and the catalytic reaction mechanisms of various metalloenzymes^13^. Overall, these cluster studies, provided they have been set up properly, yield relatively accurate activation energies that are consistent with experimental rate constants. The calculations are also able to reproduce product distributions well and give reliable geometries and electronic and spectroscopic properties that can be linked to experiments.

A consequence of the larger model size accessible today is that the application space now is wider. It has, for example, been demonstrated that it is possible to quite accurately reproduce enantioselectivity in enzymes and determine the factors controlling it^14^. The cluster approach has also been used as a predictive tool for the engineering of proteins to give them novel functions and selectivities. In an early example, a cluster model study on the nonheme iron enzyme *S*-mandalate synthase predicted how to engineer the substrate-binding pocket in order to obtain *R*-mandalate products instead, which was confirmed by experimental mutations^15^. Very recently, cluster calculations on the mechanism and origins of enantioselectivity of acyltransferase from *Mycobacterium smegmatis* assisted in the design of variants with very high selectivities for unnatural substrates^16^.

## Discussion

In general, the cluster method is highly robust and works particularly well for enzymes with small substrates. However, the cluster model is often dependent on the accuracy of the starting enzyme crystal structure, which needs to be correctly folded and should ideally have possible cofactors and substrates or substrate-analogs bound in the same conformation as its active form. If this structure is not in the active state or major folding changes happen upon substrate binding or during the catalytic cycle of the enzyme, it cannot be modeled. Similarly, large conformational movements and allosteric effects are challenging to incorporate in cluster models. One can in these cases usually resort to molecular dynamics simulations to obtain a better starting structure.

While the cluster approach produces relatively accurate reaction energies and barriers, it has difficulties reproducing absolute p*K*_a_ values and redox potentials, due to long-range effects related to the change of the overall charge of the cluster model. In these cases, one can use some experimental value to calibrate the cluster energies and obtain mechanistic insights^13^.

Despite these challenges, we believe that the cluster model approach is the method of the future, with ample opportunities for scientific research. The cluster models are usually easy to set up due to the use of 100 s of atoms as compared to 1000 s in the quantum mechanics/molecular mechanics (QM/MM) models. They suffer less from convergence problems during the calculations.

It is a robust scheme that minimizes the user-based errors that are often part of set-up procedures. The results are reproducible with different methods, software packages, starting conditions and give small deviations between the results. Importantly, the cluster approach is computationally less expensive than, e.g., QM/MM and related methods. This allows the user to examine many alternative reaction mechanisms in a relatively short time.

## Outlook

In what directions is the field likely to develop in the future? In our opinion, even if the computational resources will allow, it is not obvious that the models will grow much larger than today’s size. Often the calculations converge with size, and expanding the model further does not necessarily give more insight. Large models will lead to problems with local minima, which would require sampling and thus many more calculations. The cluster approach will also be combined with other techniques, such as empirical valence bond and free energy perturbation techniques to ask new kinds of questions. Work in these directions has already started to appear^17,18^.

As can be understood from the discussion above, we are big proponents of the cluster approach. In our opinion, it should be the method of choice for studying enzymatic reaction mechanisms. It has many advantages, and we hope that this Comment will inspire more groups to use it.
